# Fatigue in patients with head and neck cancer undergoing radiation therapy: a prospective study*

**DOI:** 10.1590/1518-8345.2813-3168

**Published:** 2019-08-19

**Authors:** Juliana Maria de Paula Avelar, Adriana Cristina Nicolussi, Bruna Francielle Toneti, Helena Megumi Sonobe, Namie Okino Sawada

**Affiliations:** 1 Universidade de São Paulo, Escola de Enfermagem de Ribeirão Preto, Centro Colaborador da OPAS/OMS para o Desenvolvimento da Pesquisa em Enfermagem, Ribeirão Preto, SP, Brasil.; 2 Estácio, Centro Universitário Estácio de Ribeirão Preto, Ribeirão Preto, SP, Brasil.; 3 Universidade Federal do Triângulo Mineiro, Departamento de Enfermagem, Uberaba, MG, Brasil.

**Keywords:** Nursing, Radiotherapy, Fatigue, Cancer, Quality of Life, Head and Neck Neoplasms, Enfermagem, Radioterapia, Fadiga, Câncer, Qualidade de Vida, Neoplasias de Cabeça e Pescoço, Enfermería, Radioterapia, Fatiga, Cancer, Calidad de Vida, Neoplasias de Cabeza y Cuello

## Abstract

**Objective:**

to identify the frequency of fatigue and domains affected in patients with head and neck cancer undergoing radiation therapy, at the beginning, middle and end of treatment.

**Method:**

longitudinal and prospective study of quasi-experimental design, involving 60 patients with head and neck cancer. It should be highlighted that this article will address only the data of the Control Group. The dependent variables were collected through interview, using the revised Piper Fatigue Scale, which is a multidimensional instrument that assesses global, behavioral, affective and sensory/psychological domains. Data analysis was based on absolute and relative frequencies.

**Results:**

there was a predominance of males, age group between 41-60 years, low level of education and in regular use of alcohol and cigarettes. All domains in the fatigue scale had their scores increased, presenting median values of greater magnitude in Time 2 and Time 3, when compared to the Time 1 values, indicating an increase in fatigue levels during radiation therapy.

**Conclusion:**

fatigue increased in the course of the radiation therapy, having all domains affected. Therefore, its evaluation throughout the treatment is important, as fatigue is a common and debilitating symptom on cancer patients.

## Abstract (pt)

**Objetivo:**

identificar a frequência do sintoma de fadiga e domínios afetados nos pacientes com câncer de cabeça e pescoço em tratamento radioterápico, no início, meio e final do tratamento.

**Método:**

estudo com delineamento quase experimental de corte longitudinal e prospectivo, envolvendo 60 pacientes com câncer de cabeça e pescoço. Destaca-se que este artigo somente abordará os dados do Grupo Controle. As variáveis dependentes foram coletadas por meio de entrevista, utilizando a Escala de Fadiga de Piper – revisada, cujo instrumento multidimensional avalia os domínios global, comportamental, afetivo e sensorial/psicológico. A análise dos dados baseou-se em frequências absolutas e relativas.

**Resultados:**

houve predomínio do sexo masculino, faixa etária de 41-60 anos, baixa escolaridade e em uso regular de álcool e cigarro. Todos os domínios da escala de fadiga tiveram seus escores aumentados, apresentando valores medianos de maior magnitude em Tempo 2 e Tempo 3, quando comparados aos valores em Tempo 1, indicando aumento nos níveis de fadiga no decorrer do tratamento radioterápico.

**Conclusão:**

o sintoma de fadiga aumentou no decorrer do tratamento radioterápico, tendo todos os domínios afetados, com isso, ressalta-se a importância da avaliação ao longo do tratamento, por se tratar de um sintoma frequente e debilitante aos pacientes oncológicos.

## Abstract (es)

**Objetivo:**

identificar la frecuencia del síntoma de la fatiga y los dominios afectados en los pacientes con cáncer de cabeza y cuello en tratamiento radioterápico, al inicio, medio y fin del tratamiento.

**Método:**

estudio con diseño cuasiexperimental de corte longitudinal y prospectivo, que contó con la participación de 60 pacientes con cáncer de cabeza y cuello. Se resalta que este artículo se limitará a abordar los datos del Grupo Control. Las variables dependientes se recopilaron por medio de entrevista, y la fatiga se valoró con la Escala de Fatiga de Piper (revisada), cuyo instrumento multidimensional valora los dominios global, comportamental, afectivo y sensorial/psicológico. El análisis de los datos se basó en frecuencias absolutas y relativas.

**Resultados:**

hubo predominio del sexo masculino, franja etaria entre 41 y 60 años, baja escolaridad y uso de alcohol y tabaco de forma regular. Todos los dominios de la escala de la fatiga tuvieron sus puntuaciones aumentadas, y presentaron valores medianos de mayor magnitud en Tiempo 2 y Tiempo 3, cuando se comparan con los valores en Tiempo 1, lo que indica un aumento en los niveles de fatiga en el transcurso del tratamiento radioterápico.

**Conclusión:**

el síntoma de fatiga aumentó durante el tratamiento radioterápico, y todos los dominios se vieron afectados, con lo que se resalta la importancia de realizar la valoración a lo largo del tratamiento, por tratarse de un síntoma frecuente y que debilita a los pacientes oncológicos.

## Introduction

Cancer is the second leading cause of death in the United States, and is considered a major public health problem nowadays. Among the ten most prevalent types of cancers, those in the oral cavity and pharynx occupy the eighth place, with estimate of 51,540 new cases for 2018, being 37,160 new cases in men and 14,380 new cases in women^1^.

The term head and neck cancer (HNC) refers to a heterogeneous group of neoplasms affecting the upper aerodigestive tract, and has as predominant histological type the squamous cell carcinoma, which comprises about 90% of the cases^2^.

HNC affects regions that are responsible for basic functions such as breathing, swallowing and verbal communication. Complications in these regions resulting from cancer and its treatment may lead to mutilation and physiological changes, as difficulties chewing, dysphagia, aspiration, changes in speech and aesthetic changes that negatively compromise the physical and psychosocial aspects of these patients^3-4^.

The most common therapeutic modalities for this type of cancer are the surgery, with or without reconstruction, the radiation therapy and the chemical therapy, which can be applied exclusively or concurrently. To choose a treatment, several factors are evaluated, such as metastasization, location and size of the tumor, with the purpose of preserving the organs, the functionality and the aesthetic issues^5^.

Radiation therapy is the most common treatment for HNC. It is used in approximately 80% of cases, with the aim of restricting the reproductive potential of cancer cells. Despite the advantage in relation to surgery as to the organ preservation, radiation therapy is also associated with numerous adverse events, since the radiation is not restricted to tumor cells, and thus, the normal cells of adjacent tissues are also affected during the treatment and can result in adverse local and widespread events^6-7^.

According to the literature, the most prevalent adverse local events of radiation therapy for HNC are: mucositis, xerostomia, secondary infections, radiation caries, trismus, dysgeusia and osteoradionecrosis^6^. And among the systemic adverse events, fatigue is most frequent symptom, associated with the radiation therapy^8^.

Fatigue is one of the most cited symptoms in the literature when considering cancer patients, and is also one of the most common side effects accompanying radiation therapy^9^. It affects from 50% to 90% of HNC patients undergoing radiation therapy. The most common factors associated with the symptom are those related to the disease itself and treatment, such as stress, decreased hemoglobin levels during treatment, weight loss, and activation of proinflammatory cytokines resulting from radiation therapy^8-10^.

Cancer-related fatigue (CRF) is a very common and debilitating symptom to the patient, being reported as an overwhelming state of exhaustion, of greater intensity and longer duration than the typical fatigue, and may have implications on therapeutic decisions, such as the interruption of therapy or dose reduction^10^. In addition, CRF is a multidimensional phenomenon that negatively affects the physical, cognitive, emotional and social domains, interfering in activities of daily living and in the course of the patient’s treatment^11^.

A study indicates the difficulty and uncertainty of the patient in reporting the symptom and barriers on the part of the health team involving lack of screening, diagnosis and treatment of the symptom, which despite being a serious and complex clinical problem, can be treated by allopathic and non-allopathic means, thus providing a better quality of life to the patient^12^.

The changes caused by the HNC linked to side effects from treatment can make the patient hopeless toward the situation experienced. In this context, a systematic review with meta-analysis found evidence that nursing intervention has a positive effect in the feeling of hope^13^. Thus, we believe that supplementary nursing interventions assist in the physical and emotional aspects of the cancer patient.

In this context, this research is appropriate considering that fatigue is multidimensional and can affect physical, psychological/emotional and social issues^12^; thus, it is believed that the nurse has an important role for patients undergoing oncological treatment. Also, to adopt an evaluation during radiation therapy for acknowledging the affected domains can subsidize the implementation of strategies that mitigate the fatigue. Specifically, the aim was to identify the frequency of the fatigue and the domains affected in the studied sample at the beginning, middle and end of radiation therapy.

## Method

This is a longitudinal and prospective study of quasi-experimental design conducted at the Radiation Therapy Center in a university hospital in the State of São Paulo – Brazil, where the ambulatory care is provided through consultations, examinations, treatment and follow-up of adult patients with cancer from the Brazilian Unified Health System (SUS), in the regions of Ribeirão Preto city.

This research is part of the study “Relaxation with guided imagery: influence on fatigue and health-related quality of life in patients with head and neck cancer during radiotherapy treatment”, whose general objective was to evaluate the effectiveness of Integrative and Complementary Practices (ICP) of relaxation and guided imagery as a strategy proposed for reducing fatigue and improving health-related quality of life.

In the present study, only the Control Group (CG) was analyzed in three moments – beginning, middle and end of the radiation therapy – to emphasize the affected domains. In this way, only the procedures of the CG will be described.

The target population of the study consisted of patients with HNC in the beginning of radiation therapy. To meet the objectives of the study, convenience sample with intentional allocation for each of the groups – Intervention Group and Control Group (IG and CG) – was used. In this sense, the sample of this study consisted of 60 patients, all belonging to the Control Group.

The estimated effect size, taking into consideration the global PIPER Scale, was equivalent to 0.63 (95%CI: 0.22-1.03) for Cohen’s d indicator. When considering the sample size in IG and CG, the power (1-β) to detect differences in one-tailed tests was equivalent to 0.70.

Inclusion criteria were: age above 18 years; HNC diagnosis; and in the beginning of radiation therapy. Exclusion criteria were: patients with some difficulty in understanding simple questions, such as date of birth, address, day of the week and current city. Questions were elaborated and applied by the researcher and, in the event of one or more wrong answers, the patient was excluded from the study.

Data collection was performed from March 2015 until March 2017. The patients answered questionnaires in the beginning – Time 1 (baseline, T1), middle – Time 2 (T2) and end – Time 3 (T3) of the radiation therapy. The researcher collected the data by individual interview in a private location.

In T1, the Sociodemographic and Clinical Characterization Questionnaire and the revised Piper Fatigue Scale (rPFS) were applied. In T2 and T3, only the revised Piper Fatigue Scale (rPFS) was applied. There was no lost to follow-up by death or withdrawal from participating in the study.

The rPFS adapted to the Brazilian audience^14^ consists of 22 items that comprise three dimensions/domains: behavioral dimension (items 2 to 7), affective dimension (items 8 to 12) and sensory/psychological dimension (items 13 to 23). Each item has a numerical scale ranging from 0 to 10. The total score is calculated by the average of all items of the instrument (items 2 to 23) and the scores of the dimensions were calculated by the average of the items in each dimension. The total score and its dimensions are described on a numerical scale from 0 to 10, given that the higher the score, the greater the indication of fatigue.

The response variables, whether dependent or of outcomes, consisted in the results of the global PIPER evaluation and its behavioral, affective and sensory/psychological domains, measured at the beginning (T1), middle (T2) and end (T3) of the radiation therapy.

The following variables that were either independent or adjusted for possible confounding effect were analyzed: sex (both sexes); age (grouped by age groups: 18-20, 21-40, 41-60, 61-80); level of education (classified as: incomplete elementary school, complete elementary school, incomplete high school, and complete high school); occupation (classified as: retiree, bricklayer/carpenter/painter, homemaker, unemployed and others); origin (classified as: Ribeirão Preto, State of São Paulo and other); religion (classified as: no preference, Catholic, Evangelical, Spiritualist); marital status (classified as: married, single, widowed and other); use of alcohol (classified as: yes, never, stopped for less than 1 year, stopped for 1 year or more); use of cigarette (classified in: yes, never, stopped for less than 1 year, stopped for 1 year or more); anatomical site of the tumor (classified as: oropharynx, larynx, oral cavity, hypopharynx, nasopharynx and salivary glands); histologic diagnosis (classified as: missing information, squamous cell carcinoma, adenoid cystic carcinoma); surgery procedure (ranked: yes, no); type of surgery (ranked: no surgery, biopsy, removal of tumor/nodule and surrounding tissue, partial removal, total removal); and staging of the disease. The staging norms followed the classification of the Union for International Cancer Control (UICC)^15^, classified as: not mentioned, I, II, III and IV.

The information collected were stored and tabulated electronically. The answers regarding the collection instrument were encoded and stored in Microsoft Excel 2010 spreadsheets. All information were typed twice in different moments and independently. Disparate values/codes were corrected, having as basis the data collection instrument. Subsequently, all spreadsheets were exported to the Stata version 13.2 application, being unified in a database source with all the information related to both study groups (IG and CG). However, this study used the information related to the CG.

The data analysis was held using absolute and relative frequencies to describe the main characteristics of the study participants. Box plot graphs were built with the aim of describing distribution details of the scores from the global PIPER scale and its respective domains, in three moments of the test application (T1, T2 and T3). To compare the averages of the PIPER scale scores, in three moments of application, the paired Student’s t-test (repeated measures) was used. A significance level of α=5% was adopted.. All analyses were held on Stata application, version 13.2.

The research project was approved by a Research Ethics Committee, and the patients’ identities were maintained in secrecy, according to resolution no. 466/2012 of the National Health Council, under the Protocol no. 26984314.9.0000.5393. Participants were informed about the objectives of the study, signed an informed consent form and received a copy of this form.

## Results

Sixty subjects who met the inclusion criteria were included. Most participants were male 53 (88.33%); 28 (46.67%) were in the age group 41-60 years, 37 (61.67%) were classified as having low level of education, 28 (46.67%) made regular use of alcohol, and 40 (66.67%) made use of cigarette. The variables of the participants are shown in Table 1.

**Table 1:** – Sociodemographic and behavioral characterization of participants. Ribeirão Preto, SP, Brazil, 2015-2017

Variables	N	N(%)
**Sex**		
Female	07	11.67
Male	53	88.33
**Age groups**		
18-20 years	02	3.33
21-40 years	27	45
41-60 years	28	46.67
61-80 years	03	5
**Level of education**		
Incomplete elementary school	37	61.67
Complete elementary school	12	20
Incomplete high school	5	8.33
Complete high school	6	10
**Occupation**		
Retiree	25	41.67
Bricklayer/carpenter/painter	15	25
Homemaker	5	8.33
Unemployed	1	1.67
Others	14	23.33
**Origin**		
Ribeirão Preto	21	35
State of São Paulo	38	63.33
Another State	1	1.67
**Marital status**		
Married	43	71.67
Single	4	6,67
Widow/widower	11	18.33
Other	2	3.33
**Religion**		
No preference	6	10
Catholic	46	76.67
Evangelical	6	10
Spiritualist	2	3.33
**Use of alcohol**		
Yes	28	46.67
Never	10	16.67
Stopped ≤ 1 year	13	21.67
Stopped > 1 year	9	15
**Use of cigarette**		
Yes	40	66.67
Never	5	8.33
Stopped ≤ 1 year	9	15
Stopped > 1 year	6	10

In relation to the clinical characterization of the participants, the anatomical site of highest incidence was the oropharynx (30%), followed by the larynx (26.67%) and the oral cavity (23.33%). The most common histological type was the squamous cell carcinoma (96.67%), and the majority of patients (51.67%) had no surgical procedure to remove the tumor and presented advanced staging (IV–45%) (Table 2).

**Table 2:** – Clinical characterization of participants. Ribeirão Preto, SP, Brazil, 2015-2017

Variables	N	N(%)
**Anatomical site of tumor**		
Oropharynx	18	30
Larynx	16	26.67
Oral cavity	14	23.33
Hypopharynx	8	13.33
Nasopharynx	3	5
Salivary glands	1	1.67
**Histologic diagnosis**		
Information missing from the chart	1	1.67
Squamous cell carcinoma	58	96.67
Adenoid cystic carcinoma	1	1.67
**Underwent surgery**		
Yes	29	48.33
No	31	51.67
**Type of surgery**		
None	31	51.67
Biopsy	1	1.67
Removal of tumor/nodule/adjacency	9	15
Partial removal	5	8.33
Total removal	14	23.33
TNM stages*		
Information missing from the chart	10	16.67
I	1	1.67
II	6	10
III	16	26.67
IV	27	45

*TNM: Tumor (T), Node (N) and Metastasis (M).

**Figure 1:**
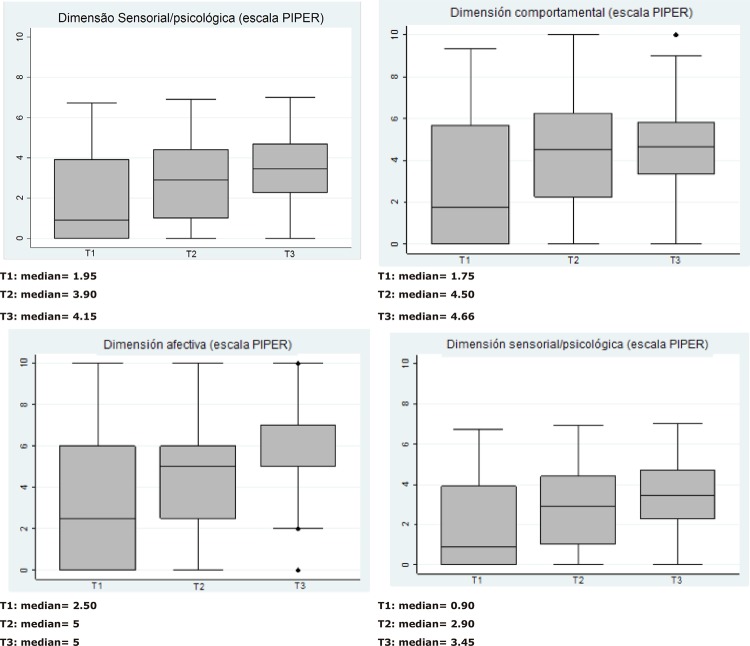
– Box plots of the PIPER scale (global score and its respective domains) in three moments of application (T1, T2 and T3). Ribeirão Preto, SP, Brazil, 2015-2017

According to Figure 1, the global scores and their respective domains presented median values of greater magnitude in T2 and T3, when compared to the T1 values, indicating an increase in fatigue levels during radiation therapy. The affective dimension was the only measure that remained with similar median values in T2 and T3, indicating stability of the score on this dimension.

In relation to multiple comparisons of the PIPER scale and its respective domains, Table 3 shows that all the differences of averages were negative (less than zero) and the respective tests indicated statistically significant differences, confirming an increase in fatigue levels during radiation therapy.

**Table 3:** – Comparison of the averages of scores of the PIPER scale and its respective domains in three moments of application (T1, T2 and T3). Ribeirão Preto, SP, Brazil, 2015-2017

Fatigue scale and domains	Student’s t-test*					
	Baseline			Measures after		
	T1-T2 Average difference	T1-T3 Average difference	T2-T3 Average difference	T1-T2 t (p value^†^)	T1-T3 t (p value^†^)	T2-T3 t (p value^†^)
global PIPER	-1.112	-1.650	-0.537	-5.127 (0.000)	-6.177 (0.000)	-3.552 (0.000)
Behavioral	-1.344	-1.866	-0.522	-4.939 (0.000)	-5.845 (0.000)	-2.995 (0.002)
Affective	-1.336	-2.093	-0.756	-4.260 (0.000)	-5.599 (0.000)	-3.653 (0.000)
Sensory/psychological	-0.883	-1.330	-0.446	-5.431 (0.000)	-5.827 (0.000)	-2.868 (0.002)

*Paired Student’s t-test; degrees of freedom=59; †p value≤0.05

## Discussion

Study participants presented as prevalent sociodemographic characteristics: male sex (88.33%), age group between 41-60 years (46.67%), low level of education/incomplete elementary school (61.67%), retirees (41.67%), and in regular use of alcohol (46.67%) and tobacco (66.67%). These findings corroborate those presented in the national and international literature about HNC patients undergoing radiation therapy. This literature shows that the prevalent characteristics are male sex, age above 40 years, low level of education as well as regular use of alcohol and cigarettes^2,7,16^.

The results for participants’ clinical characterization are also in line to the scientific literature. Studies show that the prevalent anatomical sites of cancer are the oropharynx as well as the oral cavity, and that 90% of histologic diagnoses consist of squamous cell carcinoma in advanced staging^2,16-18^.

Twenty-eight (46.67%) patients indicated regular use of alcohol and 40 (66.67%) indicated use of cigarettes, even during the radiation therapy. Other studies also identified a high prevalence of HNC patients who maintained a regular use of these substances during the oncological treatment^16-17^.

The use of alcohol and cigarettes along with oncological treatment results in a negative impact on the response of the latter, and the use of these substances after diagnosis and during treatment can be a risk factor for a secondary malignancy^17^. In addition, studies indicate that alcohol consumption is also a prognostic factor for this type of cancer, i.e. the heavy alcoholist presents a worse disease prognosis compared to non-heavy alcoholists that have stopped consuming alcohol^19-20^.

Given this scenario, it is worth mentioning that since the 1980s Brazil has been deploying, through the National Cancer Institute (INCA), a set of national actions that constitute the National Tobacco Control Program (PNCT), which aims to reduce the prevalence of smokers and, consequently, the morbidity and mortality rates related to tobacco use^21^. Furthermore, it is important to highlight the health professionals’ participation in public policies, with emphasis on the promotion of population education on anti-tobacco programs with the purpose of raising awareness of smokers to stop smoking.

This study shows that from the 60 participants, 43 (71.67%) are in advanced staging of the disease (III, IV) and, of these, 29 (48.33%) combined the radiation therapy with the surgery, which is consistent with the literature for this audience, which claims that radiation therapy is considered the standard modality for initial stage cancers and, in more advanced cases, the association of radiation therapy with surgery and/or chemical therapy is indicated^5,22-24^.

CRF is evidenced by the literature as a serious clinical problem and one of the most frequent and debilitating symptoms that affect HNC patients undergoing radiation therapy. In addition, it is responsible for the main causes of psychological disturbances, reduction of activities of daily living, social isolation, loss of motivation, and reduction of health-related quality of life, and may also influence negatively the conduction and adherence to treatment^25-27^.

A prospective study held with 40 patients with nasopharyngeal cancer aimed to evaluate the fatigue levels during radiation therapy and the possible causes of the symptom. As a result, the study identified that 60% of patients had acute fatigue during treatment, which persisted after its term. The research associated as possible causes for fatigue induced by radiation the toxicity caused by the treatment, as the increased production of pro-inflammatory cytokines [tumor necrosis factor-α (TNF-α) and Interleukin 1-β of the hippocampus, for example]^8^.

Corroborating the previous study, another recent study conducted in the United States with HNC patients also detected the presence of inflammatory markers, demonstrating that the presence of these markers due to cellular toxicity caused by the radiation therapy is associated with one of the triggering factors of fatigue^28^.

In this sense, one can note that the etiology of the symptom is complex, and can be linked not only to the intensity of the radiation received, but also to the consequence of alterations at a cellular level, especially at signaling ways in the tumor microenvironment, as in the exacerbated release of pro-inflammatory cytokines and chemokines, for example^29-30^. Thus, the inflammatory process resulting from radiation therapy may also be one of the possible causes of fatigue^25^, besides issues involving the psychological state, as stress and nutritional changes^8-9,29-30^.

A review study corroborates that the CRF etiology is complex and multidimensional, involving several potentially aggravating and contributive elements, including factors related to the tumor itself, psychological conditions, comorbidities and side effects associated with anticarcinogenic therapies or other medicines^12^.

In this research, all domains in the fatigue scale had their scores increased, showing median values of greater magnitude in T2 and T3, when compared to the T1 values, indicating an increase in fatigue levels during radiation therapy. The affective domain was the only measure that remained with similar median values in T2 and T3, indicating stability of the score on this dimension.

The results of this study are consistent with the findings of a prospective cohort study held in Amsterdam with 458 patients, which identified a significant association between the toxicity resulting from radiation therapy with the impairments in all physical, emotional, social and role performance domains. In this way, the study identified that the effects of radiation therapy lead to an increase in the fatigue symptom and an influence in all domains assessed, negatively affecting the biopsychosocial dimensions^31^.

Another prospective study performed in Brazil with 41 HNC patients undergoing radiation therapy identified the presence and increase of fatigue in 100% of participants and, despite not having assessed independently the most affected domains, identified the increase of the average of the total score during the course of treatment for all participants^25^.

According to the National Center for Complementary and Integrative Health, CFR affects 90% of oncology patients, and may negatively influence all areas of the individual’s life, making him or her too tired to take part in activities of daily living, relationships, social events and employment. In this sense, the quality of life decreases and the impairments related to adherence and continuity of treatment increase^32^.

Considering the results of this investigation, it is emphasized the importance of early detection and evaluation of the symptom of fatigue in HNC patients undergoing radiation therapy. Thus, by identifying the domains affected, a planning can be developed along with strategies that meet the individual needs of each patient and relieve and/or reduce the symptom of fatigue.

The literature highlights allopathic and non-allopathic strategies to relieve fatigue and other symptoms caused by cancer and its treatment. Among the non-allopathic strategies, there are highlighted the Integrative and Complementary Practices (ICP), such as yoga, meditation, acupuncture, relaxation techniques (such as breathing exercises, guided imagination, and progressive muscle relaxation), *Tai Chi Chuan*, *Qi Gong*, curative treatment, hypnotherapy, among others, that are used concurrently with the conventional treatment^32^.

All these practices are focused at the integral/holistic assistance of the individual and have been widely used in cancer patients, proving to be effective in pain management and reduction of anxiety and fatigue, among other adverse events of treatment, besides improving the patients’ well-being, thus contributing in the conduction of the conventional cancer treatment^32-33^.

The limitations of this study consist of the reduced number of studies that specifically assess the affected domains for a better theoretical basis that serves as a comparison to this research. Most studies that use specific instruments to evaluate fatigue presents a value for total fatigue, or within the symptom subscale in psychometric instruments that assess the health-related quality of life^8,25,28^.

In addition, another limitation refers to the non-randomization of subjects in IG and CG, due to users’ low acceptance of the relaxation and guided imagery ICP; the reduced sample size is also mentioned because this study was carried out in a single center in Brazil.

Thus, further research with similar investigations is recommended to produce studies that will allow the identification of the frequency of fatigue and its influence on different domains (physical, emotional, social), which also influences the choice of the intervention to be adopted, which may be allopathic or not.

The findings of this study contribute to the knowledge of professionals who work providing assistance to cancer patients, in order to motivate the use of psychometric instruments for evaluation of fatigue and identification of the affected domains, because it is an effective and low-cost evaluation, which provides evidence for the implementation of strategies for relief and/or reduction of the symptom.

## Conclusion

The results show an increase of fatigue during the course of radiation therapy, allowing the inference that the symptom of fatigue caused by treatment negatively affects the patients in different domains (global, behavioral, and sensory/psychological), and can influence the everyday life and the conduction of the oncological treatment of patients.

In order to provide a holistic treatment, it is important to evaluate the fatigue levels, as well as those of the affected domains, throughout the treatment, since that, as soon as the symptom is detected, health professionals can start applying strategies for fatigue reduction, thus contributing in the conduction of conventional treatment, but also contributing to the patient’s well-being.

## References

[1] Siegel RL, Miller KD, Jemal A (2018). Cancer statistics, 2018. CA Cancer J Clin.

[2] Majid A, Sayeed BZ, Khan M, Lakhani M, Saleem MM, Rajani H (2017). Assessment and Improvement of Quality of Life in Patients
Undergoing Treatment for Head and Neck Cancer. Cureus.

[3] Pinto GP, Mont’alverne DGB (2015). Neoplasms of head and neck: impacts functional and quality of
life. Rev. Bras. Cir Cabeça Pescoço.

[4] Krebber A-MH, Van Uden-Kraan CF, Melissant HC, Cuijpers P, Van Straten A, Becker-Commissaris A (2017). A guided self-help intervention targeting psychological distress
among head and neck cancer and lung cancer patients: motivation to start,
experiences and perceived outcomes. Support Care Cancer.

[5] Cohen EE, LaMonte SJ, Erb NL, Beckman KL, Sadeghi N, Hutcheson KA (2016). CA: Cancer J Clinicians.

[6] Strojan P, Hutcheson KA, Eisbruch A, Beitler JJ, Langendijk JA, Lee AWM (2017). Treatment of late sequelae after radiotherapy for head and neck
cancer. Cancer Treat Rev.

[7] Paula JM, Sawada NO (2015). Health-related quality of life of cancer patients undergoing
radiotherapy. Rev Rene.

[8] Powell C, Schick U, Morden JP, Gulliford SL, Miah AB, Bhide S (2014). Fatigue during chemoradiotherapy for nasopharyngeal cancer and
its relationship to radiation dose distribution in the brain. Radiother Oncol.

[9] Hsiao CP, Daly B, Saligan LN (2016). The Etiology and management of radiotherapy-induced
fatigue. Expert Rev Qual Life Cancer Care.

[10] Lipsett A, Barrett S, Haruna F, Mustian K, O’donovan A (2017). The impact of exercise during adjuvant radiotherapy for breast
cancer on fatigue and quality of life: A systematic review and
meta-analysis. Breast.

[11] Lavoy EC, Fagundes CP, Dantzer R (2016). Exercise, inflammation, and fatigue in cancer
survivors. Exerc Immunol Rev.

[12] Koornstra RH, Peters M, Donofrio S, van den Borne B, de Jong FA (2014). Management of fatigue in patients with cancer – A practical
overview. Cancer Treat Rev.

[13] Li P, Guo YJ, Tang Q, Yang L (2018). Effectiveness of nursing intervention for increasing hope in
patients with cancer: a metaanalysis. Rev. Latino-Am. Enfermagem.

[14] Mota DDCF, Pimenta CAM, Caponero R (2012). Fatigue in colorectal cancer patients: prevalence and associated
factors. Rev. Latino-Am. Enfermagem.

[15] International Union Against Cancer (United States) (2004). TNM Classification of malignant th tumors.

[16] Felippu AWD, Freire EC, Arruda Silva R, Guimarães AV, Dedivitis RA (2016). Impact of delay in the diagnosis and treatment of head and neck
cancer. Braz J Otorhinolaryngol.

[17] Schiller U, Inhestern J, Burger U, Singer S, Guntinas-Lichius O (2016). Predictors of post-treatment smoking and drinking behavior of
head and neck cancer survivors: results of a population-based
survey. Eur Arch Otorhinolaryngol.

[18] Rigoni L, Bruhn RF, De Cicco R, Kanda JL, Matos LL (2016). Quality of life impairment in patients with head and neck cancer
and their caregivers: a comparative study. Braz J Otorhinolaryngol.

[19] Leoncini E, Vukovic V, Cadoni G, Pastorino R, Arzani D, Bosetti C (2015). Clinical features and prognostic factors in patients with head
and neck cancer: Results from a multicentric study. Cancer Epidemiol.

[20] Sawabe M, Ito H, Oze I, Hosono S, Kawakita D, Tanaka H (2017). Heterogeneous impact of alcohol consumption according to
treatment method on survival in head and neck cancer: a prospective
study. Cancer Sci.

[21] Ministério da Saúde (BR), Instituto Nacional de Câncer, Organização Pan-Americana da Saúde (2011). Pesquisa especial de tabagismo – PETab: Relatório Brasil / Organização
Pan-Americana da Saúde.

[22] Galbiatti ALS, Padovani JA, Maníglia JV, Rodrigues CDS, Pavarino ÉC, Goloni-Bertollo EM (2013). Head and neck cancer: causes, prevention and
treatment. Braz J Otorhinolaryngol.

[23] Demian NM, Shum JW, Kessel IL, Eid A (2014). Oral surgery in patients undergoing chemoradiation
therapy. Oral Maxillofacial Surg Clin N Am.

[24] Vinés VE, Orellana GMJ, Bravo MC, Jofré PD (2017). Management of head and neck cancer: when, why, and to whom give
radiotherapy. Rev Otorrinolaringol Cir Cabeza Cuello.

[25] Sawada NO, Paula JM, Sonobe HM, Zago MM, Guerrero GP, Nicolussi AC (2012). Depression, fatigue, and health-related quality of life in head
and neck cancer patients: a prospective pilot study. Support Care Cancer.

[26] Murphy BA, Deng J (2015). Advances in Supportive Care for Late Effects of Head and Neck
Cancer. J Clin Oncol.

[27] Ebede CC, Jang Y, Escalante CP (2017). Cancer-Related Fatigue in Cancer Survivorship. Med Clin N Am.

[28] Xiao C, Beitler JJ, Higgins KA, Conneely K, Dwivedi B, Felger J (2016). Fatigue is associated with inflammation in patients with head and
neck cancer before and after intensity-modulated radiation
therapy. Brain, Behav Imm.

[29] Saligan LN, Olson K, Filler K, Larkin D, Cramp F, Yennurajalingam S, Escalante CP, del Giglio A, Kober KM, Kamath J, Palesh O, Mustian K (2015). The biology of cancer-related fatigue: a review of the
literature. Support Care Cancer.

[30] Lopes LC, Olson K, de Omena Bomfim E, Pereira-da-Silva G, Nascimento LC, de Lima RA (2016). Translational research and symptom management in oncology
nursing. Br J Nurs.

[31] Langendijk JA, Doornaert P, Verdonck-de Leeuw IM, Leemans CR, Aaronson NK, Slotman BJ (2008). Impact of late treatment-related toxicity on quality of life
among patients with head and neck cancer treated with
radiotherapy. J Clin Oncol.

[32] National Center for Complementary and Integrative Health
(NCCIH) (2017). Complementary, Alternative, or Integrative Health: What’s In a
Name?.

[33] Tabatabaee A, Tafreshi MZ, Rassouli M, Aledavood SA, AlaviMajd H, Farahmand SK (2016). Effect of Therapeutic Touch in Patients with Cancer: a Literature
Review. Med Arch.

